# CMR and Percutaneous Treatment of Pulmonary Regurgitation: Outreach the Search for the Best Candidate

**DOI:** 10.3390/life13051127

**Published:** 2023-05-04

**Authors:** Francesca Baessato, Peter Ewert, Christian Meierhofer

**Affiliations:** 1 Department of Cardiology, Regional Hospital S. Maurizio, 39100 Bolzano, Italy; 2 Congenital Heart Disease and Pediatric Cardiology, German Heart Center Munich, 80636 Munich, Germany

**Keywords:** pulmonary regurgitation, congenital heart disease, cardiovascular magnetic resonance, cardiac computed tomography, percutaneous pulmonary valve implantation

## Abstract

Performance of cardiovascular magnetic resonance (CMR) in the planning phase of percutaneous pulmonary valve implantation (PPVI) is needed for the accurate delineation of the right ventricular outflow tract (RVOT), coronary anatomy and the quantification of right ventricular (RV) volume overload in patients with significant pulmonary regurgitation (PR). This helps to find the correct timings for the intervention and prevention of PPVI-related complications such as coronary artery compression, device embolization and stent fractures. A defined CMR study protocol should be set for all PPVI candidates to reduce acquisition times and acquire essential sequences that are determinants for PPVI success. For correct RVOT sizing, contrast-free whole-heart sequences, preferably at end-systole, should be adopted in the pediatric population thanks to their high reproducibility and concordance with invasive angiographic data. When CMR is not feasible or contraindicated, cardiac computed tomography (CCT) may be performed for high-resolution cardiac imaging and eventually the acquisition of complementary functional data. The aim of this review is to underline the role of CMR and advanced multimodality imaging in the context of pre-procedural planning of PPVI concerning its current and potential future applications.

## 1. Introduction

Pulmonary valve replacement (PVR) is indicated in selected cases to treat the chronic volume overload on the right cardiac chambers secondary to severe pulmonary regurgitation (PR). The pulmonary valve (PV) consists of three semilunar cusps, one anterior and two posteriors, which connect through the fibrous interleaflet triangles to the right ventricular outflow tract (RVOT). PR may occur as a sequela of the surgical repair of congenital heart diseases (CHD), such as tetralogy of Fallot (TOF), where surgical correction consists of closing the ventricular septal defect and widening the pulmonary outflow tract [1]. Other causes of PR may be related to congenital PV defects, valve prolapse, endocarditis, rheumatic disease, carcinoid syndrome, or secondary to pulmonary artery (PA) dilatation. Chronic PR determines volume overload in the right ventricle (RV), which progressively dilates, potentially leading to RV systolic dysfunction, relevant tricuspid regurgitation, ventricular arrhythmias, and heart failure [2].

Percutaneous pulmonary valve implantation (PPVI) has emerged in recent decades and has shown earlier treatment promise in PR compared to surgery before irreversible RV failure occurs [3]. PPVI can help to avoid the complications related to cardiopulmonary bypasses and sternotomies and has a favorable reduction in hospital stay and patient recovery time when compared to surgical PVR. Recent studies have demonstrated encouraging long-term results of PPVI in terms of infective endocarditis, the need for reintervention and overall survival [4].

An extensive clinical and instrumental pre-procedural workup is essential to select the optimal timing and possible candidates for PPVI. A meticulous preprocedural analysis of the RVOT and PA anatomy is needed, as percutaneous prosthetic valves are available in defined types of shape and dimension, while the anatomy and sizes of the RVOT are very heterogeneous. This could also help in avoiding possible PPVI-related complications such as stent fractures, device embolization, conduit rupture, and coronary artery compression [5].

Different non-invasive imaging modalities are applied in the routine clinical practice for PPVI planning [6,7]. Among these, two-dimensional (2D) transthoracic echocardiography represents the first-line imaging modality for the assessment of post-operative cardiac anatomy and function thanks to its accessibility, low costs, and high temporal and spatial resolution. However, echocardiography is limited by poor acoustic windows, operator dependency, and the fact that the presence and grading of PR are mostly evaluated visually. Two-dimensional echocardiography also allows a relatively restricted field of view with incomplete three-dimensional (3D) visualization of the complex structure of the RV, especially in adult patients [8].

Therefore, second-level imaging with cardiovascular magnetic resonance (CMR) or cardiac computed tomography (CCT) is fundamental for PPVI planning. Principal advantages of CMR are that it can provide both anatomical and hemodynamic information, with accurate quantification of RV volumes and function, precise quantification of PR, and a 3D anatomical definition of the RVOT, PA and coronary arteries. Unlike echocardiography, CMR allows unrestricted views on cardiac and extracardiac structures, such as conduits, and is independent from geometric assumptions for volume quantification. In the last few years, CCT has been increasingly applied before PPVI as an alternative to CMR. Its principal role relies on high-resolution 3D evaluation of cardiovascular anatomy and the delineation of coronary arteries. Despite anatomical data, recent evolution technology allows CCT to acquire functional data related to blood flows and ventricular volumes [9,10].

A multimodality imaging approach is fundamental to optimally plan complex percutaneous procedures over the PV [11]. Among all the different non-invasive imaging modalities, CMR still remains the current standard for serial PPVI planning due to the absence of ionizing radiation and its excellent quantification of PR and RV volume overload as well as its good depiction of the thoracic and coronary anatomy.

The aim of this review is to underline the role of CMR in the context of pre-procedural planning of PPVI concerning its current and potential applications in the future.

## 2. Right Ventricular Volume Overload and Clinical Indications for PPVI

**Figure 1:**
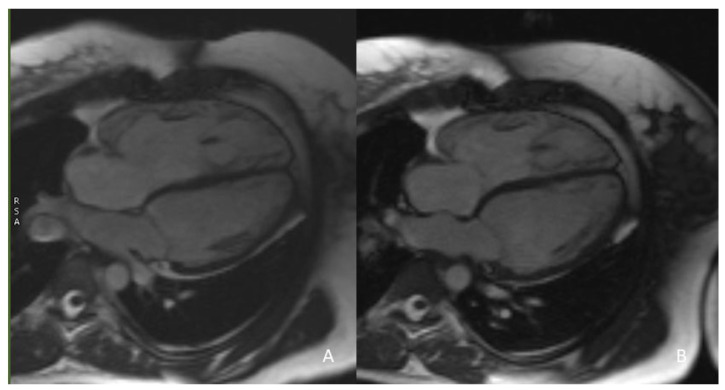
(**A**) 32-year-old patient, male. Significant reduction in RV dilatation is evident after PPVI (**B**) in comparison to the previous CMR study before the procedure (**A**), as highlighted in this 4-chamber view cine.

CMR is currently considered the gold standard for ventricular volume quantification and the assessment of systolic function. A reduction in RV volume overload has been shown in CMR studies after successful PPVI treatment; an example is given in Figure 1.

In recent decades, numerous attempts have been made to identify reasonable cut-offs of RV dilatation in terms of end-diastolic volume (RVEDV) and end-systolic volume (RVESV) for PVR indication in patients with relevant PR, as, in most cases, patients can remain clinically asymptomatic for years despite relevant PR and severe RV dilatation and/or initial deterioration of its contractile function [12,13]. Follow-up with CMR studies is needed for seriate RV volume quantification [14]. Thanks to its high reproducibility, CMR represents an ideal tool to monitor RV dimensions and pump function over time to identify the best timing for intervention [15]. Currently, no defined clinical biomarker has been confirmed to identify patients with PR who should undergo PPVI.

In asymptomatic patients, indications for PVR are based essentially on the entity and trend of RV dilatation and dysfunction.

The most recent 2020 guidelines of the European Society of Cardiology (ESC) for the management of adult CHD consider PPVI as the preferred treatment in TOF patients with no native RVOT, if technically feasible (class of recommendation I, level of evidence C). In asymptomatic repaired-TOF patients, the presence of severe PR and/or RVOT are considered indications for PPVI, with at least one of the following: decrease in exercise capacity; progressive RV dilatation with indexed RVEDV ≥ 160 mL/m^2^, and/or indexed RVESV ≥ 80 mL/m^2^, and/or the development of at least moderate tricuspid regurgitation; progressive RV contractile dysfunction (recommendation IIa) [16]. Similar indications in asymptomatic patients are given by the 2018 American guidelines for the management of adults with CHD [17]. Of note, the calculation of RV volumes can be slightly variated by tracing the papillary muscles and RV trabeculations and excluding them from the blood pool, as well as by assessing volumes in axial or short-axis slices [18]. In symptomatic patients, a class I indication for PVR is given when moderate or severe RV-PA conduit stenosis and/or regurgitation is present, with no specific recommendations for PPVI over surgical treatment [16]. PPVI can be performed in cases of dysfunctional surgically implanted RV-PA conduits with or without bioprosthetic valves with at least a 16 mm diameter [19]. In patients with dysfunctional native RVOT, PPVI could be performed in selected patients, although no standard agreement exists for either surgical or percutaneous treatment [20].

## 3. CMR for PPVI Feasibility: RVOT, PA and Coronary Anatomy

Essential requirements for a successful PPVI procedure are a precise pre-procedural sizing and assessment of the RVOT and PA anatomy as well as the origin and course of the coronary arteries.

### 3.1. PPVI Valves

The Melody valve (Medtronic, Dublin, Ireland) was the first implanted PPVI valve and is available for RVOTs up to a 22 mm diameter. Currently, the principal concern is represented by patients with large RVOTs (>31 mm), as device embolization may occur. Very recently, new valves have been provided for RVOTs with large diameters, but in some cases PPVI may still not be feasible. Eventually, pre-stenting of the RVOT with one or more stents may be needed to reduce the dimensions of a large RVOT and optimize the landing zone of the implanted valve [21]. The Edwards SAPIEN 3 valve (Edwards Lifesciences, Irvine, CA, USA) is, like the Melody valve, balloon-expandable. It is available for 20 mm, 23 mm, 26 mm, and 29 mm valve sizes and can be used up to a 30 mm RVOT diameter, including pre-stenting [22]. The PULSTA valve (TaeWoong Medical Co., Gimpo-si, Republic of Korea) is anatomically suitable for RVOT diameters up to 28 mm, while the Venous *p*-valve (Venus MedTech, Hangzhou, China) can be used for large RVOTs up to 36 mm in diameter [23,24,25]. An asymmetric self-expanding stent with larger proximal and distal ends characterizes the Harmony valve (Medtronic), which already has FDA approval and could be used in different RVOT types with very large diameters (≥40 mm) [26].

### 3.2. RVOT and Coronary Arteries

Another important aspect is the RVOT anatomy, which may affect high heterogeneity in CHD patients.

A previous CMR study identified five different RVOT types in patients referred for PPVI. Among these, the pyramidal type was the commonest, but most frequently treated surgically due to the high risk of device dislocation [27]. In contrast, the RVOT straight, funnel, convex and hourglass types seemed to be the most suitable for PPVI [28].

**Figure 2:**
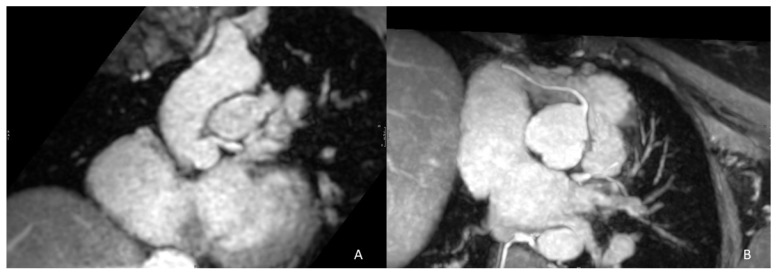
Examples of an unfavorable coronary anatomy for PPVI. Extreme proximity of the left main coronary artery to the RVOT (<1 mm) has contraindicated percutaneous pulmonary intervention (**A**). (**B**) shows an acute take-off angle (<90°) of the proximal right coronary artery which is an issue for successful PPVI.

In addition to RVOT morphology and size, pre-procedural representation of the origin and course of the coronary arteries is fundamental to hypothesize potential coronary compression in the placement of the new valve, especially when this is planned to be oversized in comparison to the existing PV. The course of the left main and descending anterior coronary artery in proximity to the RVOT as well as a take-off angle ≤90° of the left main and right coronary arteries may increase the PPVI failure rate or even contraindicate it (Figure 2) [29]. Final invasive evaluation during cardiac catheterization is performed to cross-check data with a selective coronary angiography during balloon inflation in the RVOT to evaluate possible coronary compression during valve placement [30].

### 3.3. Technical CMR Sequences

**Figure 3:**
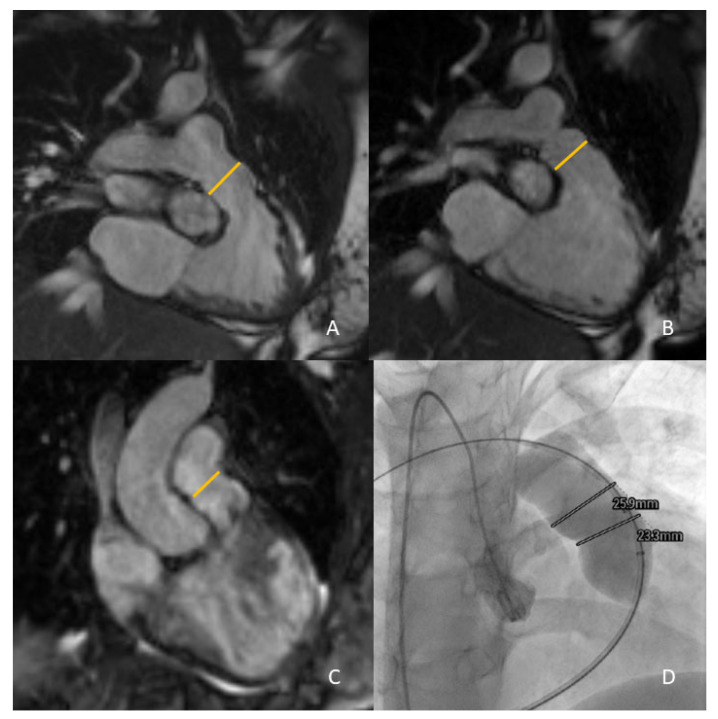
Measurement of the RVOT by CMR for PPVI feasibility and valve sizing: 2D cine views at end-systole and end-diastole ((**A**) and (**B**), respectively), and 3D whole heart imaging sequences (**C**). Final check of CMR data with invasive angiography (**D**).

For the RVOT anatomy, different anatomical CMR sequences can be performed, such as contrast-enhanced MR angiography (ceMRA) and 3-dimensional (3D) whole-heart sequences (Figure 3).

3D whole-heart sequences are preferable in children as they do not require breath-holding or a contrast medium [31,32]. Additionally, they can provide imaging of the coronary arteries, especially of the proximal course which is at the highest risk for compression during the PPVI procedure [33]. A breathing navigator prospectively allows the acquisition of images only at a defined phase of the respiratory cycle, thus reducing breathing artefacts. Through a retrospective ECG trigger, scanning is set at a specific phase of the cardiac cycle, either systole or diastole, which results in a significant reduction in blurring artefacts due to cardiac structures and vessel motion.

The main limitations of 3D whole-heart sequences are their high susceptibility to metallic devices (stents or protheses), relatively long acquisition times, and RVOT measurement variations of up to 5 mm between systole and diastole, as demonstrated in a recent study [34]. ceMRA is a fast 3D gradient-echo sequence for vessel assessment, which requires a contrast medium (gadolinium). It is performed in a single breath-hold, and in most cases with no ECG triggering [35]. This may result in the imprecise measurement of vessel dimensions being affected by their movements through the cardiac cycle [36].

The different timings of image acquisition between 3D whole-heart and ceMRA inevitably introduce a clear difference in RVOT measurement. Ferrari et al. compared RVOT measurements by ceMRA and 3D steady-state-free-precession (SSFP) sequences at end-systole (ES) and end-diastole (ED) in a retrospective study of 31 CHD patients with moderate to severe PR, in native or patched RVOTs and significant RV dilatation. This study demonstrated that 3D SSFP diameters at ES were significantly higher than those taken at ED and with ceMRA. In this study, ES diameters with whole-heart imaging also showed the best agreement with cardiac catheterization data [34]. A study by Leonardi et al. evidenced that RVOT measurements by 3D SSFP sequences acquired at ES were able to identify the ideal candidates for PPVI when compared to sequences acquired in mid-diastole in operated TOF patients.

Moreover, ES data showed the best correlation with cardiac catheterization to quantify RVOT dilatation. In contrast, mid-diastolic measures seemed to underestimate the RVOT size when compared to angiographic data [37]. In another study, Ebel et al. showed that the maximum and effective RVOT diameters acquired in 3D SSFP at ES had the best agreement with invasive balloon sizing and presented an optimal image quality and reproducibility against ceMRA [33].

## 4. CMR Protocol for PPVI

**Figure 4:**
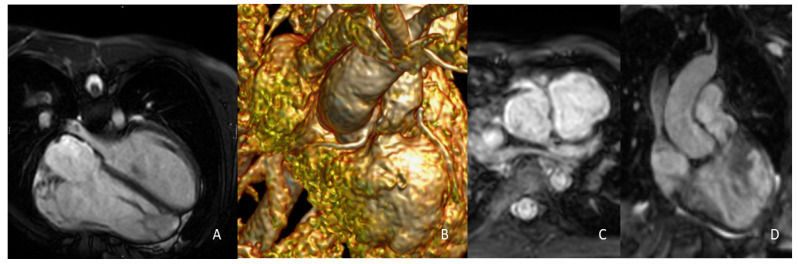
Fundamental CMR sequences for PPVI planning. The 2D cine SSFP sequences for volumetric assessment and systo-diastolic RVOT measurement (**A**). The 3D whole-heart sequences for coronary (**B**,**C**) and static RVOT (**D**) imaging. ceMRA may be used as an alternative for assessment of pulmonary anatomy. CMR: cardiovascular magnetic resonance, PPVI: percutaneous pulmonary valve implantation, 2D: two-dimensional, SSFP: steady-state free-precession, RVOT: right ventricular outflow tract, 3D: three-dimensional, ceMRA: contrast enhanced magnetic resonance angiography.

At our institution, all potential candidates for PPVI underwent a complete CMR protocol for the evaluation of the PV, RVOT and PA anatomy, quantification of PR and biventricular volumes, and assessment of coronary anatomy (Figure 4).

Our experience was based on the performance of CMR studies using a 1.5-tesla (T) (MAGNETOM Avanto^®^, Siemens Healthineers, Erlangen, Germany) or a 3 T scanner (Philips Ingenia Elition, Boston, MA, USA), both with a cardiac phased array coil placed on the thorax. A standardized CMR dataset was set for all patients to minimize acquisition times, along with a CMR study that can be performed in approximately 30–40 min. The presence of well-trained technicians and direct on-site supervision by an experienced cardiac imager are fundamental to check acquired images and eventually add further sequences or scanning planes if needed. The CMR study protocol for PPVI candidates essentially consists of 2D cine SSFP in multiple cardiac planes, and 3D SSFP for anatomical imaging and phase contrast (PC) sequences for flow quantification (Table 1).

Retrospectively ECG-gated, breath-hold, 2D cine SSFP sequences are typically used for qualitative and quantitative evaluation of the RV. These are acquired in the 4-chamber view and in 2 perpendicular RVOT views to assess the RVOT expansion during the cardiac cycle. Eventually, a RVOT/main PA view could be added to address the RVOT simultaneously with the PV and main pulmonary trunk. In the presence of metal artefacts due to a previous stent or conduit, gradient echo cine images can be added for higher image quality. An axial stack is obtained from the coronal and sagittal localizers to cover the heart from just below the diaphragm to the pulmonary bifurcation. These axial slices allow a dynamic evaluation of the RVOT and are used for volume analysis. For volume quantification, endocardial contours of both ventricles are manually traced at ES and ED, excluding papillary muscles and trabeculations, which are considered part of the cardiac mass. Previous studies have shown high reproducibility and accuracy of CMR volume analysis in patients with CHDs at our center [38,39].

For anatomical assessment, 3D SSFP navigator sequences are commonly acquired at ED to optimize coronary artery imaging. Measurements are taken with a double oblique technique in a multi-planar reconstruction mode. A distance <1 mm between the RVOT and the course of a coronary artery, usually the descending anterior, is considered a higher risk for PPVI failure. A balloon sizing during cardiac catheterization is usually performed for final evidence.

Concerning the RVOT, measurements are taken at the proximal, mid, and distal level of the main, right and left PA. In addition, the smallest diameter of the RVOT should be addressed as a potential landing zone for the new valve, as well as the length of the main PA that has to be covered by the stent which determines the use of either the PULSTA or Harmony valve.

Flow data are obtained with 2D PC images, which currently give more rapid results than the most recent 4D sequences in both acquisition and post-processing phases. Standard planes for flows are acquired in the ascending aorta, and the main, right and left PA. This allows direct calculation of the regurgitant volume and regurgitant fraction of the PV and differential lung perfusion [40]. Fundamental aspects for optimal PR quantification with PC imaging are the adequate planning of acquisition planes, due to often complex and distorted RVOT anatomy, and the setting of a proper velocity encoding rate to avoid aliasing artefacts due to high velocity regurgitation jets or concomitant valve stenoses. Table 2 specifies the different cut-offs for PR severity when assessed with CMR.

Contrast medium is not usually administered and is only limited to restricted cases where additional LGE or angiographic sequences are needed.

## 5. Role of Cardiac CT

Although CMR is the most used non-invasive imaging modality to evaluate potential candidates for PPVI, CCT has shown an increasing role in the noninvasive imaging of CHD patients thanks to its excellent spatial resolution and short acquisition times (usually a single breath-hold) [41]. CCT provides the acquisition of a 3D dataset of the entire thoracic and cardiac structures that can be reconstructed in multiplanar views. Its principal limit is represented by the risk of ionizing radiation, but this has been significantly reduced by latest-generation CCT scanners and dose-reduction technologies such as dual-energy and dynamic CCT [42].

In the context of PPVI evaluation, the role of high-resolution cardiovascular imaging by CCT principally consists of an anatomical assessment. CCT can clearly demonstrate the anatomy of the RVOT and distribution of the PA [43] and is considered the gold-standard for noninvasive coronary imaging. Moreover, CCT is superior to CMR for the visualization of position and function of endovascular stents, presence of RVOT pseudoaneurysms, or tissue calcifications along RV-PA conduits, which are also important aspects before PPVI to guide the procedure.

However, no consensus exists on CCT measurements that should be used to best assess valve size and patients’ suitability. A recent retrospective study by Curran et al. evaluated CCT parameters such as the minimum and maximum diameter of the PV (pulmonary valve), the cross-sectional area and the circumference-derived diameter in patients who had already undergone PPVI. Of these, the cross-sectional area and the circumference-derived diameter were best associated with the real PV anulus size [44]. Radiation exposure and the possibility of the measurement of PA diameters, either in systole or diastole in a single examination (with the exception of 4D CCT studies), also make CCT not ideal for the longitudinal assessment of PR patients.

Schievano et al. evidenced the wide variability of RVOT/PA morphology, size and dynamics through 4D CCT examinations which provided 3D deformation data in 12 patient candidates for PPVI. When compared to standard static images by 3D CMR, a high disagreement was evidenced, mainly due to complex anatomy and deformation forces [45]. In another study, 4D data with CCT were used to generate a bespoke patient-specific valve. The positioning and stabilization of the device were demonstrated using a 3D prototype model derived from the patient’s anatomy [28]. CCT-derived data were also used by Jolley et al. to virtually assess the position of a Harmony valve in the RVOT and PA using a specific 3D software. Although CCT images helped in ideally reproducing the procedure, no reliable assessment of RVOT expansion after device placement was possible [46]. In a retrospective study, a 3D hybrid technique with the fusion of the CMR and CCT datasets was applied in candidates for PVR. The authors demonstrated how the creation of a 3D volume-rendered reconstruction through CMR and CCT images facilitated the invasive procedure with reduced interventional times and radiation exposure [47].

Although its principal role consists of a pure anatomical assessment, CCT can assess biventricular volume and function quantification as well as the evaluation of RV contraction abnormalities, and may be used as an alternative to CMR when this is contraindicated [9,10]. For volume quantification, a retrospective ECG gating with data acquisition along the entire cardiac cycle is required. However, this determines a definitively higher radiation exposure than commonly performed anatomical CCT scans with a prospective ECG acquisition [48]. Table 3 compares advantages and disadvantages of CCT versus CMR concerning PPVI planning.

## 6. Advanced 3D Methods for PPVI Planning

In last few years, 3D data, principally through CMR and CCT studies, have satisfied the clinical need to overcome the standard 2D assessment of cardiac structures to better appreciate complex intracardiac relationships in CHD patients. This has hugely facilitated a patient-tailored approach in surgical and interventional planning [49].

3D printing can provide patient-specific physical 3D reconstructions of the entire heart or of specific regions of interest. Acquired 3D CMR (whole-heart or ceMRA) or multidetector CCT images are transformed into 3D surface files after an accurate post-processing analysis, and sent to the printer for the final result. Good image quality with high contrast between cardiac and vessel structures, low noise and high spatial resolution are fundamental for adequate 3D printing [50]. Important advantages of 3D printing are represented by the direct and 3D visualization of complex anatomical structures with the opportunity of a “hands-on” and personalized PPVI simulation [51,52]. In patients with severe PR and a complex RVOT anatomy before PPVI, 3D-printed models of the RVOT and PAs may significantly facilitate valve choice, family counselling and communication with young patients [53,54]. Although supporting evidence for the clinical use of 3D printing has been given in several case reports and few randomized studies [55], further prospective studies with cost–benefit and long-outcome analyses are needed to define the use of 3D-printed cardiac models in the routine clinical practice of CHDs.

Recently introduced virtual and augmented reality technologies have offered 3D visualization and simulation of complex congenital defects in real-time [56]. Moreover, patient-specific computational fluid dynamic models and the application of artificial intelligence have shown great potential in the prediction of PPVI-related complications, as complementary tools to standard CMR and CCT imaging [57]. However, limited evidence is known for clinical application of such appealing technologies in CHD patients so far.

## 7. Conclusions

Essential goals of CMR studies before PPVI are the definition of the origin, course, and take-off angle of the coronary arteries, the RVOT morphology and dimensions, the grading of PR severity, and the quantification of RV volumes and ejection fraction. All these data can be achieved through standard 2D and 3D CMR sequences and should be routinely addressed for patients’ selection and to optimize PPVI planning. Innovative imaging approaches, such as 4D flow CMR sequences and the application of artificial intelligence algorithms, may further impact our future workflow in CMR scanning. However, these are still mainly restricted to the research area and further studies are warranted to assure their full integration into routine clinical practice.

## Figures and Tables

**Table 1:** Study protocol for CMR in patients undergoing possible PPVI. PROs and CONs refer to advantages and disadvantages related to each CMR sequence, respectively.

CMR Sequence	Standard Views	PROs and CONs
2D SSFP cine	4-chamber Axial (from diaphragm to PA) RVOT RVOT-PA Right and left PA	PROs - Axial slices for volume quantification: 0 mm gap, 6 mm (children) and 8 mm (adults) slice thickness - ECG triggering: RVOT measurements at both end-systole and end-diastole (dynamic sequences) CONs - Breath-hold - High susceptibility to metal artefacts (stents or prothesis): ev. gradient-echo cine sequences are less affected
3D whole-heart	Comprehensive view of thoracic and cardiac anatomy	PROs - Free-breathing - No contrast-medium - Coronary imaging - ECG triggering: RVOT measurements at end-systole or end-diastole (static sequences) CONs - Long acquisition times - High susceptibility to metal artefacts
ceMRA	Right cardiac chambers RVOT PA	PROs - Single breath-hold - Fast acquisition times CONs - Contrast medium - No coronary imaging - No ECG triggering (blurring artefacts)

CMR: cardiovascular magnetic resonance, PPVI: percutaneous pulmonary valve implantation, 2D: two-dimensional, PA: pulmonary arteries, RVOT: right ventricular outflow tract, ECG: electrocardiogram, 3D: three-dimensional, ceMRA: contrast enhanced magnetic resonance angiography.

**Table 2:** Definition of PR severity by CMR.

	Mild	Moderate	Severe
Regurgitant fraction (%)	0–15	16–30	>30
Regurgitant volume (ml)	0–20	21–40	>40

PR: pulmonary regurgitation, CMR: cardiovascular magnetic resonance.

**Table 3:** PROs and CONs of CMR and CCT scanning in potential PPVI patients.

	PROs and CONs
CMR	PROs - No ionizing radiation (ideal for serial follow-up and children) - Anatomical and functional data within the same examination; gold standard for quantification of ventricular volumes and pulmonary regurgitation - Contrast medium required only for angiographic sequences when needed CONs - Longer acquisition times - Limited or contraindicated in certain subsets of patients (es. metal devices)
CCT	PROs - Fast acquisition times (usually a single breath-hold) - High spatial resolution with excellent imaging of coronary arteries and thoracic anatomy - Less susceptible to metal artefacts CONs - Ionizing radiation - Contrast medium required - Principally anatomical data (functional data possible only with retrospective ECG gating) - Either systolic or diastolic measurements (both measurements possible only with 4D CCT protocols)

CMR: cardiovascular magnetic resonance, CCT: cardiac computed tomography, ECG: electrocardiogram, 4D: four-dimensional.
